# Fine-Tuned Machine Learning Classifiers for Diagnosing Parkinson’s Disease Using Vocal Characteristics: A Comparative Analysis

**DOI:** 10.3390/diagnostics15050645

**Published:** 2025-03-06

**Authors:** Mehmet Meral, Ferdi Ozbilgin, Fatih Durmus

**Affiliations:** 1 Department of Neurosurgery, Private Erciyes Hospital, Kayseri 38020, Türkiye; m.meral@erciyeshastanesi.com.tr; 2 Department of Electrical and Electronic Engineering, Giresun University, Giresun 28200, Türkiye; 3 Department of Electrical and Electronic Engineering, Ondokuz Mayıs University, Samsun 55270, Türkiye; fatih.durmus@omu.edu.tr

**Keywords:** brain disease, Parkinson, optimization, machine learning, classification, diagnostic

## Abstract

**Background/Objectives**: This paper is significant in highlighting the importance of early and precise diagnosis of Parkinson’s Disease (PD) that affects both motor and non-motor functions to achieve better disease control and patient outcomes. This study seeks to assess the effectiveness of machine learning algorithms optimized to classify PD based on vocal characteristics to serve as a non-invasive and easily accessible diagnostic tool. **Methods**: This study used a publicly available dataset of vocal samples from 188 people with PD and 64 controls. Acoustic features like baseline characteristics, time-frequency components, Mel Frequency Cepstral Coefficients (MFCCs), and wavelet transform-based metrics were extracted and analyzed. The Chi-Square test was used for feature selection to determine the most important attributes that enhanced the accuracy of the classification. Six different machine learning classifiers, namely SVM, k-NN, DT, NN, Ensemble and Stacking models, were developed and optimized via Bayesian Optimization (BO), Grid Search (GS) and Random Search (RS). Accuracy, precision, recall, F1-score and AUC-ROC were used for evaluation. **Results**: It has been found that Stacking models, especially those fine-tuned via Grid Search, yielded the best performance with 92.07% accuracy and an F1-score of 0.95. In addition to that, the choice of relevant vocal features, in conjunction with the Chi-Square feature selection method, greatly enhanced the computational efficiency and classification performance. **Conclusions**: This study highlights the potential of combining advanced feature selection techniques with hyperparameter optimization strategies to enhance machine learning-based PD diagnosis using vocal characteristics. Ensemble models proved particularly effective in handling complex datasets, demonstrating robust diagnostic performance. Future research may focus on deep learning approaches and temporal feature integration to further improve diagnostic accuracy and scalability for clinical applications.

## 1. Introduction

Parkinson’s Disease (PD) is a long-term neurodegenerative disorder resulting from the degeneration of dopamine-producing neurons in the central nervous system, impacting nearly 10 million individuals worldwide. PD prevalence is estimated at 1% among individuals over 60 and increases to 4% in those over 85, with men being 1.5 times more likely to develop the disease than women. PD seriously affects motor functions, cognitive abilities, and quality of life. Approximately 60,000 new cases are reported worldwide each year, and the incidence of the disease has been increasing markedly in recent years. In 2019, PD caused 329,000 deaths and 5.8 million disability-adjusted life years lost [1]. Symptoms usually appear slowly; motor symptoms include bradykinesia, tremors, and postural instability, while non-motor symptoms include depression, anosmia, and dementia [2,3,4]. Conventional diagnostic techniques are often applied in later disease stages, underscoring the need for non-invasive approaches that enable early detection and timely intervention [5].

PD symptoms include voice disorders, loss of balance, and tremor [6,7]. Studies indicate that over 90% of PD patients exhibit vocal abnormalities, including dysphonia, dysarthria, monotony, and hypophonia [8,9,10]. Therefore, voice disorders are usually one of the first symptoms noticed in individuals with PD [11]. As a non-invasive and straightforward technique, voice analysis presents a viable tool for monitoring disease progression [12,13]. Various voice tests, such as continuous phonations and speech texts, have been developed for this purpose [14].

Machine learning and artificial intelligence applications span various fields such as energy [15,16] and health [17,18]. Specifically, machine learning techniques have demonstrated significant potential in detecting and classifying PD. Max A. Little et al. [19] introduced the Pitch Period Entropy (PPE) metric, an effective approach for identifying dysphonia in noisy and uncontrolled environments, achieving an accuracy of 91.4% on 195 audio recordings from 31 individuals. Resul Das [20] evaluated the performance of various classifiers and reported that Neural Networks (NNs) yielded the highest accuracy, with a rate of 92.9%. Another study employing genetic algorithms and k-nearest neighbor (k-NN) demonstrated that feature selection enhanced classification accuracy to over 85% [21]. Similarly, B.E. Sakar et al. [22] achieved an accuracy of 85% on audio data collected from 40 individuals using Support Vector Machines (SVMs) and k-NN algorithms. Studies involving classifiers such as random trees, SVMs, and logistic regression reported accuracy rates reaching up to 100% [23]. Furthermore, in a comparative analysis of SVMs and NNs, SVMs demonstrated superior performance with an accuracy of 93.33% [24]. Collectively, these findings underscore the importance of effective feature selection and the application of appropriate machine learning algorithms in achieving high classification accuracy for the early diagnosis of PD.

The goal of this study is to contribute to the literature by combining Chi-Square-based feature selection with a comparison of three hyperparameter optimization techniques, GS, RS and BO, to establish which of these is the most appropriate for classifying PD. This paper provides a systematic approach of enhancing the accuracy and time of applying machine learning for PD diagnosis through comparison of the optimization techniques and feature selection techniques. The contribution of this study to the literature can be outlined as follows:

- It provides a comprehensive analysis of feature selection, machine learning classifiers, and hyperparameter optimization techniques (GS, RS, and BO), demonstrating their impact on the accuracy and efficiency of PD diagnosis using vocal characteristics.
- It shows that Ensemble Learning methods outperform individual classifiers in PD diagnosis, and among them, the proposed Stacking-based framework achieves the highest predictive performance, making it the most effective machine learning approach in this study.
- It improves model interpretability by using SHapley Additive exPlanations (SHAP) analysis to identify the most influential vocal features in PD classification, providing a clearer understanding of their diagnostic significance.

This paper is structured into five main sections. Section 1 offers the general background of PD, clarifies the current diagnostic methods, and explains how the present work helps to advance the knowledge in the area. Section 2 details the dataset, the feature selection process, the machine learning models, and the hyperparameter optimization methods. Section 3 presents the experimental results and evaluates the effect of the different optimization strategies on the classification accuracy. Section 4 discusses the findings in comparison with other studies, focusing on the advantages and disadvantages of the proposed approach. Finally, Section 5 here summarizes the main findings and recommends possible directions for future investigations.

## 2. Materials and Methods

This section describes the dataset and methods used for PD classification. It provides an overview of the data preprocessing steps, including feature selection and preparation for machine learning algorithms. The relevant methods are detailed in the following sections under specific headings.

### 2.1. Dataset

**Figure 1:**
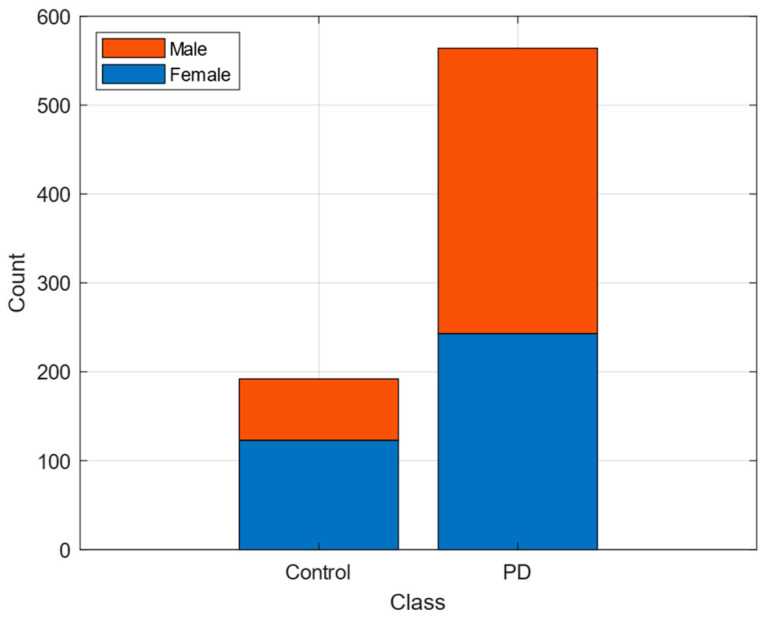
Gender distribution of samples in the dataset.

The dataset utilized in this study consists of publicly available data compiled by Sakar et al. [22,25]. It encompasses voice recordings from 188 patients diagnosed with PD and 64 healthy controls, including 107 males and 81 females, with an age range of 33 to 87 years (mean age: 65.1 ± 10.9 years). Additionally, the dataset includes recordings from a control group comprising 64 healthy individuals (23 males and 41 females) aged between 41 and 82 years (mean age: 61.1 ± 8.9 years). The bar chart in Figure 1 illustrates the gender distribution within the PD and control groups. The PD group has a notably higher number of participants compared to the control group, reflecting the higher prevalence of PD in the studied population.

The data collection process involved the use of a microphone with a sampling rate of 44.1 kHz, and each participant was instructed to sustain the phonation of the vowel /a/ three times following a physician’s examination. In total, the data contain 7568 speech recordings with 754 feature columns. Of these 754 columns, 1 corresponds to a unique identifier for each recording, 1 denotes the binary class label, and the remaining 753 columns represent diverse acoustic and signal-processing-based attributes. Information about the features is given below [25].

Baseline Features include various Jitter variants, which are employed to assess the instabilities in the oscillatory patterns of the vocal folds. This feature set quantifies cycle-to-cycle variations in the fundamental frequency and comprises five distinct parameters. Similarly, Shimmer variants are designed to assess instabilities in the oscillatory amplitude of the vocal folds, quantifying cycle-to-cycle variations in amplitude, and comprise six features. Fundamental frequency parameters assess the frequency of vocal fold vibrations and encompass five distinct features: mean, median, standard deviation, minimum, and maximum values. Harmonicity parameters focus on the increased noise components resulting from incomplete vocal fold closure associated with speech pathologies. Key metrics include the Harmonics to Noise Ratio and the Noise to Harmonics Ratio, both of which quantify the relationship between signal information and noise. Recurrence Period Density Entropy (RPDE) offers insights into the stability of vocal fold oscillations and quantifies deviations from the fundamental frequency (F0) (1 feature). Detrended Fluctuation Analysis (DFA) evaluates the stochastic self-similarity of turbulent noise and is represented by a single feature. Pitch Period Entropy (PPE) quantifies impaired control over the fundamental frequency (F0) using a logarithmic scale and is also represented by a single feature. Lastly, intensity parameters, which reflect the power of speech signals in decibels (dB), include three features: mean, minimum, and maximum intensity values.

Time Frequency Features include formant frequencies, which are amplified by the vocal tract. The first four formants are used as features (four features). Additionally, bandwidth measures the frequency range between formant frequencies, with the first four bandwidths also considered as features (four features).

Mel Frequency Cepstral Coefficients (MFCCs) measure the impact of PD on the vocal tract, separate from the influence of the vocal folds. We utilize a total of 84 MFCC features.

Wavelet Transform-Based Features use wavelet transform (WT) to quantify deviations in the fundamental frequency. This feature set includes 182 distinct features.

Vocal Fold Features begin with the Glottis Quotient (GQ), which measures the opening and closing durations of the glottis, providing insight into periodicity (3 features). The Glottal to Noise Excitation (GNE) quantifies the level of turbulent noise generated by incomplete vocal fold closure during speech (6 features). The Vocal Fold Excitation Ratio (VFER) assesses the proportion of pathological noise produced by vocal fold vibrations, utilizing concepts of nonlinear energy and entropy (7 features). Lastly, Empirical Mode Decomposition (EMD) breaks down speech signals into elementary components using adaptive basis functions, with energy or entropy values derived from these components used to quantify noise (6 features).

### 2.2. Chi-Square Test

The Chi-Square (χ^2^) test is a statistical method used to assess the relationship between categorical independent variables and a categorical or continuous dependent variable. It is particularly valuable in classification tasks, especially for feature selection. This method determines the independence hypothesis of every feature with respect to the target variable, i.e., which features are most significant. The test works by comparing the observed and expected frequencies and how much difference in these affects the target variable. At its core, the Chi-Square test comes with two hypotheses: the null hypothesis (H0), which states that independence and hence the feature is unrelated to the target variable, and the alternative hypothesis (Ha), which states that there is dependency between the two [26].

The Chi-Square test statistic is calculated using the following formula:

(1) $$\chi^{2}=\sum \frac{{\left(O-E\right)}^{2}}{E}$$

Here, *O* represents the observed values, and *E* denotes the expected values. For each category, the squared difference between the observed and expected values is divided by the expected value, and these results are summed to compute the test statistic. The resulting χ^2^ value is then compared against a critical value obtained from the Chi-Square distribution, considering the degrees of freedom and a predefined significance level (*p*-value). By conducting this comparison, the Chi-Square test facilitates the identification of features that exhibit a stronger association with the target variable, making it particularly valuable in high-dimensional datasets. The selection of the most relevant features enhances both the performance and efficiency of machine learning models.

### 2.3. Machine Learning

Support Vector Machine (SVM) is a powerful machine learning technique used for classification and regression tasks. The primary objective of SVM is to identify the optimal hyperplane that divides the data into distinct classes. This hyperplane maximizes the margin, which is the distance between the boundary and the nearest data points from each class. Generally, the SVM decision function can be written as [27]

(2) $$S\left(X\right)=\sum_{i=1}^{l}\alpha_{i}z_{i}k\left(X_{i},X\right)+b$$

where $k\left(X_{i},X\right)$ is the kernel function, b is the bias, and $\alpha_{i}$ is the coefficients to be found. It is important to note that the SVM model frequently utilizes various kernel functions, such as the linear kernel, polynomial kernel, radial basis function (RBF), and sigmoid kernel.

k-Nearest Neighbor (k-NN) is a straightforward and efficient technique for classification and regression issues. A data point’s class or value is determined by the algorithm by taking into account the labels of its *k* closest neighbors. While Euclidean distance ($d\left(x,y\right)=\sqrt{\sum {\left(x_{i}-y_{i}\right)}^{2}}$) is the most widely used distance measure, alternative metrics like Manhattan or Minkowski could be more desirable. While the average of the labels is used for regression, the majority vote of the neighbors is used for classification [28].

As a non-parametric technique, k-NN does not need any assumptions on the distribution of data and may be used in a variety of fields, including medical diagnosis, recommendation systems, and handwriting recognition. However, the value of *k* and the distance metric used determine how well the method performs. There are drawbacks as well, such as higher computing costs for big datasets or high-dimensional data [28].

Decision Tree (DT) is one of the most effective supervised learning algorithms and is used for both classification and regression problems. This algorithm learns the decision processes by building up a tree-like structure of the dataset. The DT has internal nodes that make decisions based on a single feature and branches that partition the dataset into subgroups based on these decisions. Leaf nodes contain class labels or predicted values [29].

The main advantage of DTs is that they are easy to interpret and can model both linear and nonlinear relationships. However, this method is sensitive to the risk of overfitting and its performance is usually increased with editing methods such as pruning or depth limitation. DTs are frequently preferred in various application areas such as customer segmentation, medical diagnosis and credit risk analysis [30].

Neural Network (NN) is a powerful modeling technique inspired by the biological nervous system and has a wide range of applications in machine learning. NNs learn complex relationships in data using multi-layered structures of interconnected nodes (neurons). Basically, starting from the input layer, the data are processed through hidden layers and transmitted to the output layer. Each connection is represented by weights, and the network’s learning process works to optimize these weights.

NNs are usually trained using supervised learning methods and the weights are updated using backpropagation algorithms [16]. The network has the capacity to model nonlinear relationships and forms the basis of deep learning applications. NNs show high performance in many complex problems such as image processing, natural language processing, voice recognition and financial analysis. However, training the model can require large datasets and computational resources.

Ensemble Learning is a machine learning method that integrates multiple learning algorithms to generate better and more accurate predictions. This method aims to create a more robust prediction model by compensating for the weaknesses of individual models. The principle is to achieve better performance by combining models that use different algorithms or the same algorithm with different parameters. Bagging, Boosting and Stacking are the most common Ensemble methods [31].

To train each model on a different subset of data and then average the results to make the final prediction, a technique called Bagging or Bootstrap Aggregating is employed. Boosting is a technique where a large number of weak learners are trained sequentially and each model attempts to improve on the errors made by the previous model. A new model is trained by combining several models’ outputs, and this is called Stacking. Ensemble Learning is used in a variety of application domains that demand highly accurate models, for example, financial forecasting, medical diagnoses, and text classification [32].

Stacking Learning is an Ensemble Learning method that leverages multiple base learners and a meta-learner to enhance predictive performance. Unlike Bagging and Boosting, which primarily focus on reducing variance and bias, respectively, Stacking aims to improve generalization by combining diverse model outputs in an optimal manner [33].

In the Stacking framework, multiple base models—often referred to as level-0 learners—are trained on the same dataset, each capturing different aspects of the data distribution. The predictions from these base models are then used as input features for a higher-level model, known as the meta-learner or level-1 model, which learns to aggregate these outputs effectively. This meta-model is typically a more flexible algorithm, such as a linear regression, SVM, or NN, chosen based on its ability to generalize across the base models’ predictions [34].

In this study, the machine learning methods employed for individual classification tasks were also used as base models in the Stacking framework. To ensure optimal performance, the model that achieved the best results among the individual classifiers was selected as the meta-learner.

### 2.4. Hyperparameter Optimization

Hyperparameter optimization is an important step in the development of effective machine learning models since the choice of hyperparameters is known to affect model performance considerably. In this study, three different strategies of hyperparameter optimization were applied, namely BO, GS and RS. Each method offers specific benefits in the hyperparameter space exploration and the tradeoff between the computational expense and model accuracy.

BO is a probabilistic model-based optimization algorithm that uses a surrogate model of the objective function to search the hyperparameter space. BO does not require the explicit formulation of the objective function; rather, it uses techniques such as Gaussian Processes to forecast the most promising regions of the search space based on prior evaluations. This adaptive and sequential approach helps to explore more informedly and often finds optimal hyperparameters with less evaluations than exhaustive methods [35]. Specifically, in this study, BO was very helpful in managing high-dimensional search spaces where computational resources were scarce.

GS is a classical optimization strategy that evaluates all possible combinations of hyperparameter values within given ranges systematically. This exhaustive search guarantees that the global optimum of the search parameters is discovered within the specified parameter space. However, the computational complexity of GS increases exponentially with the number of hyperparameters and the granularity of the search grid, and thus it is not suitable for models with many and complex parameters. Nonetheless, GS was used in this study to establish a broad baseline for hyperparameter optimization.

RS, on the other hand, selects hyperparameter combinations randomly from a given distribution. This method does not search through all possible combinations as GS does but instead selects points randomly, which can be advantageous over GS in high dimensional spaces [36]. By using computational resources for the broad exploration of the parameter space, Random Search often finds good hyperparameter settings with greatly reduced runtime. In this study, RS was used to support the results of BO and GS, especially when the parameter space was too large to be exhausted by exhaustive techniques. The three optimization techniques used in this study ensured a comprehensive and fair comparison of the hyperparameter configurations and thus enhanced the performance of the machine learning models used.

### 2.5. Performance Evaluation

The assessment of model performance is an essential process in determining the appropriateness of the proposed approach for PD classification. In this study, the efficiency of machine learning models optimized by three hyperparameter optimization techniques, BO, GS, and RS, is evaluated using multiple quantitative metrics to ensure a thorough analysis of the classification accuracy, robustness, and generalization capability.

To assess the classification performance, the following metrics were used: accuracy, error rate, precision, sensitivity (recall), F1-score, and AUC-ROC. All these metrics were defined mathematically as follows [37,38,39]:

- Accuracy and Error Rate

(3) $$Accuracy=\frac{TP+TN}{TP+TN+FP+FN}\times 100$$

(4) Error Rate=100−Accuracy

where *TP* and *TN* are the true positive and true negative counts, respectively, and *FP* and *FN* are the false positive and false negative counts.

- Precision

(5) $$Precision=\frac{TP}{TP+FP}\times 100$$

*Precision* reflects the proportion of true positive predictions among all positive predictions, indicating the model’s reliability in identifying relevant cases.

- Recall (Sensitivity)

(6) $$Recall=\frac{TP}{TP+FN}\times 100$$

*Recall* measures the model’s ability to correctly identify all true positive cases, an essential metric in medical applications where minimizing false negatives is critical.

- F1-score

(7) $$F1\text{-}score=2\times \frac{Precision\times Recall}{Precision+Recall}\times 100$$

The *F*1*-score* balances precision and recall, particularly useful in cases of imbalanced datasets.

- AUC-ROC

The area under the receiver operating characteristic curve (AUC-ROC) is a threshold-independent metric that assesses the balance between the true positive rate (TPR) and the false positive rate (FPR). Higher AUC values signify better discrimination between classes.

The dataset is divided into training and testing subsets using a stratified approach to maintain the class distribution in both subsets. A k-fold cross-validation technique with k = 5 is used during model training to ensure a robust performance evaluation and reduce the risk of overfitting. The hyperparameter optimization techniques (BO, GS, and RS) are applied to maximize classification performance on the training data. The optimized models are subsequently evaluated on the testing data to validate their generalization ability.

### 2.6. Proposed Methodology

**Figure 2:**
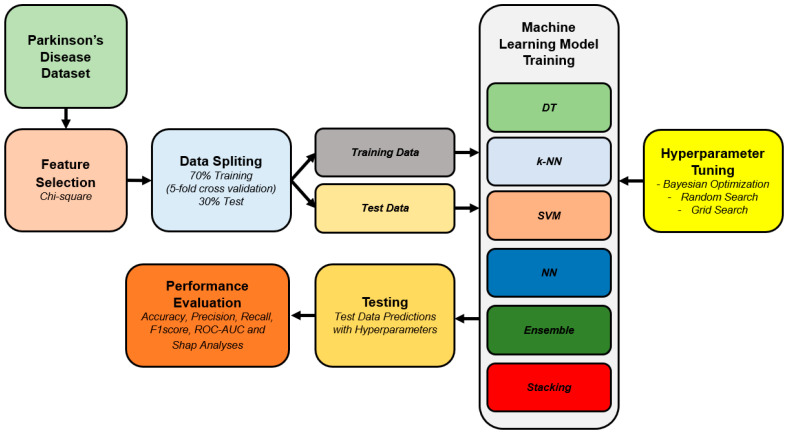
Proposed methodology.

In order to classify PD, the methodology explained below is illustrated in Figure 2. The approach proposed here starts with the dataset, which is then followed by feature selection and data partition into training and test sets. The methodology encompasses model training and hyperparameter optimization strategies, together with the comparison of multiple machine learning models, to achieve the best performance. The results are also backed up by performance metrics and SHAP analyses to make the findings more accurate and easy to interpret.

**Figure 3:**
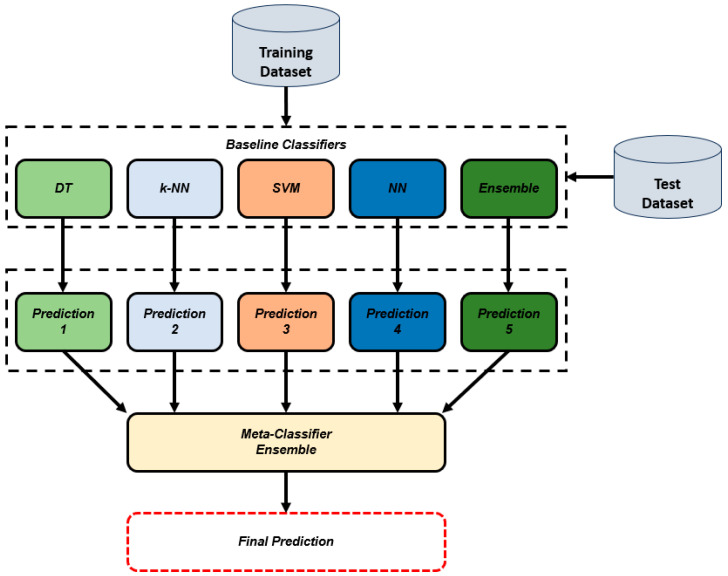
Proposed Stacking Learning method.

The dataset used in this study has 753 features. In the proposed methodology, feature selection is performed as the next step, whereby parameters with Chi-Square importance values above 20 are retained. This threshold was proposed to find a balance between the dimensionality reduction and the inclusion of features that are really important in the classification process so that computational time is not increased unnecessarily. After the feature selection is complete, the data are divided into training and testing sets at a 70:30 ratio. This division ensures that there are enough data to train the machine learning models and yet keeps a good test set to validate the models. Figure 3 shows a visualization of the proposed stack learning method. Within the scope of the study, five different ML methods are used as baseline classifiers. The Ensemble method, which gives the best accuracy value, is used as a meta-classifier.

The hyperparameters for the ML classifiers used in the study are optimized through three different optimization methods. In this context, BO, GS and RS methods are employed for looking for parameters that would produce the most favorable performance of each of the ML algorithms. The hyperparameters of the ML algorithms optimized in the search process, along with their search values, are illustrated in detail in Table 1. In this study, the default parameter ranges defined by MATLAB 2024b’s Classification Learner application were used to enable the optimization process to explore the most effective values without unnecessary constraints.

Finally, the performance evaluation phase is conducted based on the selected parameters for the training and testing data. In this phase, metric values for the prediction results, as defined by Equations (3)–(6), along with ROC curves and AUC values, are obtained. Lastly, SHAP analysis, a feature importance method, is applied to the model that demonstrated the best performance to provide interpretability and insights into the model’s predictions.

## 3. Results

In this study, vocal parameters of patients and machine learning methods with parameters adjusted using three different optimization techniques were employed to predict PD. The study, presented as a comparative analysis, utilized a computer with an Intel Core i7-13700H processor, 32 GB RAM, an 8 GB RTX 4070 GPU, and the MATLAB programming language.

Table 2 shows the ranking of the features used in the classification of PD according to their Chi-Square scores. Within the scope of the study, attributes with Chi-Square scores of 20 and below were not used in the analysis due to their low information contribution. In total, 156 features with Chi-Square scores above 20 were included in the evaluation. The threshold value of 20 was selected empirically to balance dimensionality reduction and feature importance. The wavelet transform-based features group was the most represented, with 132 features. These features, including wavelet-based energy, entropy, TKEO, and statistical measures (e.g., tqwt_TKEO_std_dec_12, tqwt_entropy_shannon_dec_12, tqwt_minValue_dec_12), played a critical role in identifying PD. The Baseline Features group comprised eight features, including Jitter (e.g., locAbsJitter, rapJitter), Shimmer (e.g., apq11Shimmer), and meanIntensity, which analyze the fundamental acoustic parameters of vocal performance. The Vocal Fold Features group included 13 features, such as Glottis Quotient (GQ_std_cycle_open) and Glottal to Noise Excitation (GNE), which measure glottal function and assess pathological noise. The Time-Frequency Features group, containing two features, focused on formant frequencies and time-frequency analyses to evaluate disease indicators. Finally, one feature from the Mel Frequency Cepstral Coefficients (MFCCs) group (mean_MFCC_2nd_coef) was included to analyze the effects of PD on the vocal tract independently.

Table 3 presents the hyperparameters obtained for different machine learning classifiers using three optimization techniques, BO, GS, and RS, in the context of PD prediction. The results highlight variability in the selected hyperparameters across methods, reflecting their unique optimization strategies. For instance, BO identified more complex configurations for DT and k-NN compared to RS, which favored simpler setups. Similarly, GS generally produced intermediate hyperparameter values, balancing model complexity and simplicity. Notably, NNs optimized via BO resulted in larger and deeper architectures, while RS identified minimal configurations. These variations underscore the influence of optimization strategies on model design and their potential impact on predictive performance. Among the Ensemble models, GentleBoost demonstrated the best overall performance, achieving approximately 2.5% higher accuracy and 1.5% higher F1-score compared to Bagging, 3% higher accuracy and 1.8% higher F1-score than LogiBoost, 1% higher accuracy and 0.6% higher F1-score than AdaBoost, and nearly 2% higher accuracy than RUSBoost. These results were obtained using Bayesian Optimization (BO); however, similar performance improvements were observed with Grid Search (GS) and Random Search (RS), indicating the consistency and robustness of the findings across different hyperparameter optimization methods.

In Table 4, various hyperparameter search strategies (BO, GS and RS) are applied to different classification algorithms (DT, SVM, k-NN, NN, Ensemble and Stacking) and evaluated on both training (validation) and testing datasets. Overall, k-NN, Ensemble and Stacking methods demonstrate consistently higher accuracy, precision, and F1-scores, alongside relatively lower error rates. In particular, GS-based Stacking and Ensemble models exhibit superior performance compared to the other approaches in the test phase, with Stacking achieving the highest accuracy across all configurations. These findings suggest that the advantageous combination of base learners in Ensemble and Stacking methods, as well as the sensitivity of k-NN to precise parameter tuning, contributes to their effectiveness. Stacking, by leveraging multiple base models and an optimal meta-learner, outperforms other Ensemble methods, demonstrating its ability to refine and aggregate predictions for improved generalization. Consequently, the reported metrics highlight the substantial impact of selecting the appropriate hyperparameter search strategy and classifier on model performance in both training and testing stages.

**Figure 4:**
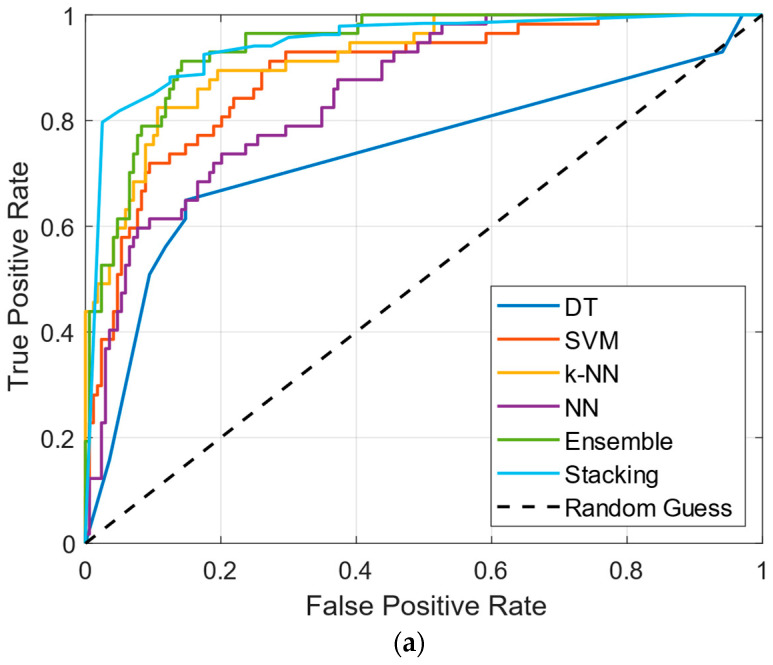

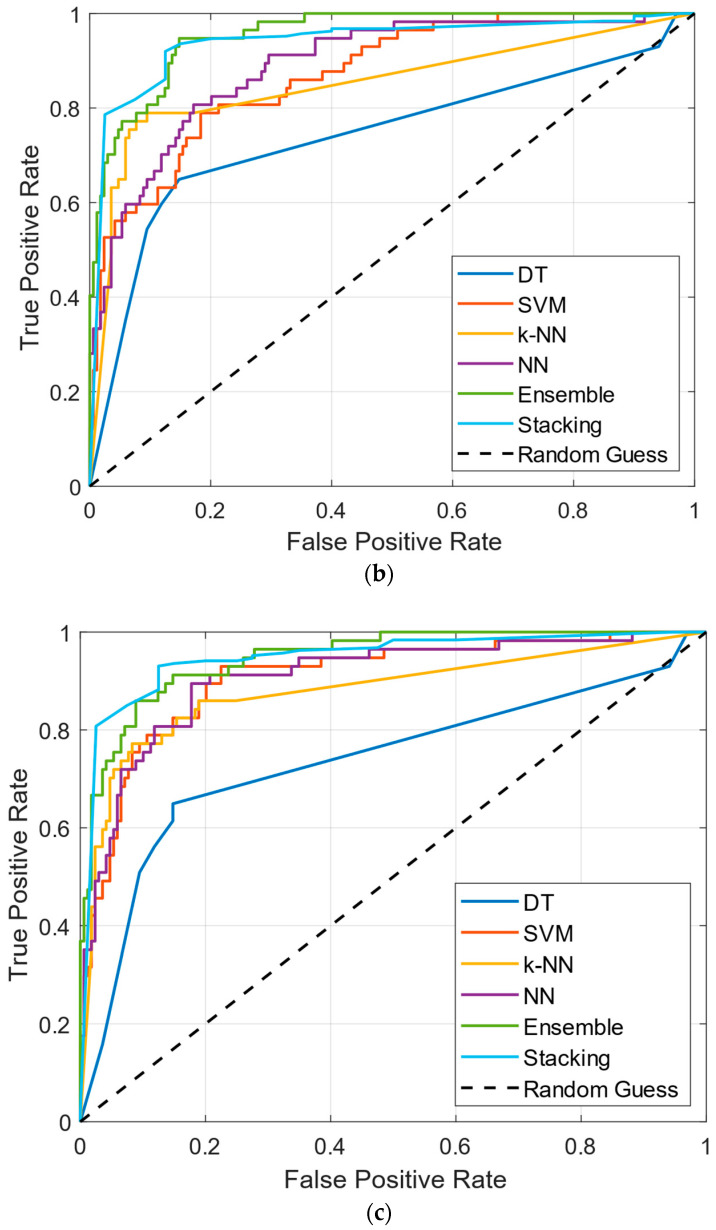
ROC curves of machine learning classifiers optimized (**a**) with BO parameters, (**b**) with RS parameters, and (**c**) with GS parameters.

Figure 4 illustrates the ROC curves for machine learning classifiers optimized using three different parameter tuning methods: (a) BO, (b) RS, and (c) GS. Across all methods, Stacking and Ensemble classifiers demonstrated superior performance, with ROC curves closely approaching the top-left corner, indicating a high true positive rate and a low false positive rate. The Stacking model consistently outperformed all other classifiers, showing the highest area under the curve (AUC) across all optimization strategies. This highlights the effectiveness of combining multiple base learners and selecting the best-performing model as the meta-learner. The NN and SVM classifiers also exhibited strong ROC curves, particularly under RS and GS methods. In contrast, the DT classifier consistently showed the lowest ROC curve performance, reinforcing its limitations in this context. The random guess line (y = x) serves as a baseline for comparison, further emphasizing the effectiveness of Stacking and Ensemble methods in PD classification.

**Figure 5:**
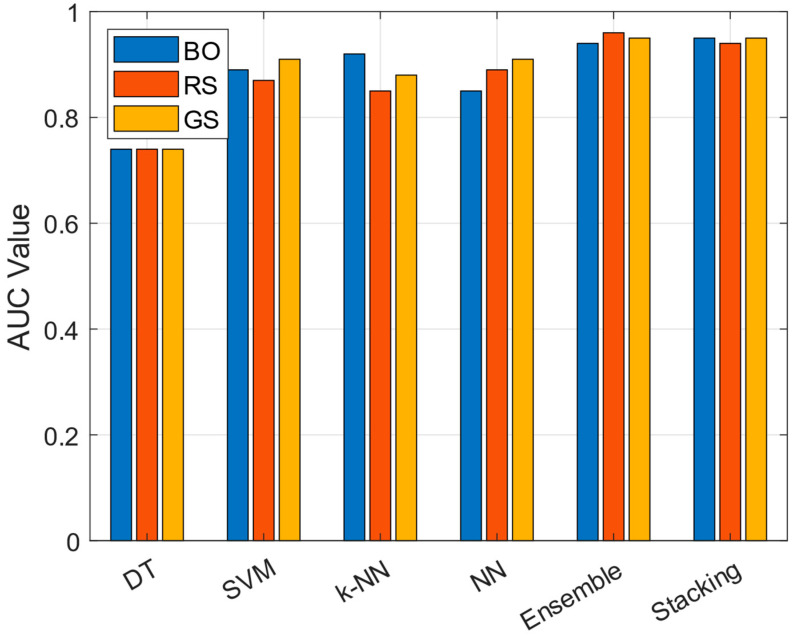
Comparison of AUC values across different models and optimization methods for PD classification.

Figure 5 compares the AUC values of different optimization methods (BO, RS, GS) applied to various models (DT, SVM, k-NN, NN, Ensemble, and Stacking). Stacking and Ensemble models demonstrated the best performance across all optimization methods, with RS-Ensemble achieving the highest AUC value of 0.96, followed closely by GS-Stacking and BO-Stacking with 0.95. GS was particularly effective for SVM, NN, and Stacking models, while BO showed strong performance with both the Ensemble and Stacking models. In contrast, the DT model consistently exhibited the lowest performance with an AUC value of 0.74 across all methods. While RS previously emerged as the most effective optimization approach due to its superior performance with the Ensemble model, the inclusion of Stacking shows that both GS and BO can yield comparable results. Overall, Stacking and Ensemble models proved to be the most successful in PD classification, highlighting the advantage of combining multiple base learners for improved predictive accuracy.

**Figure 6:**
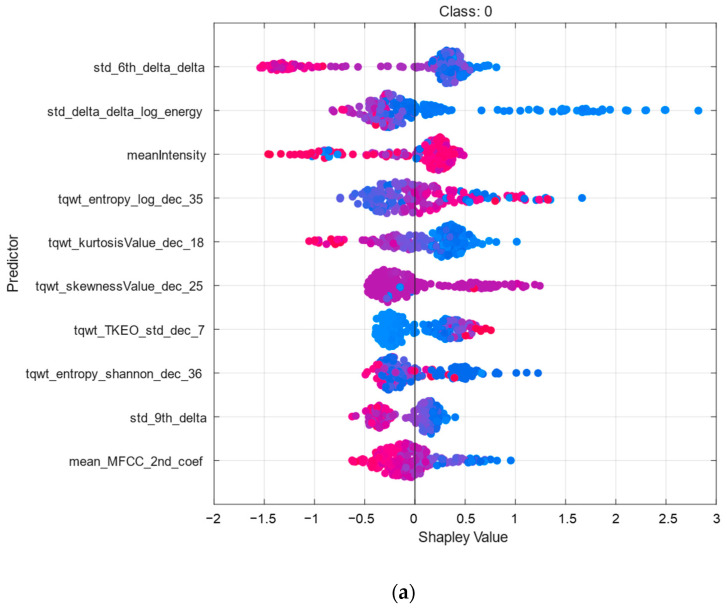

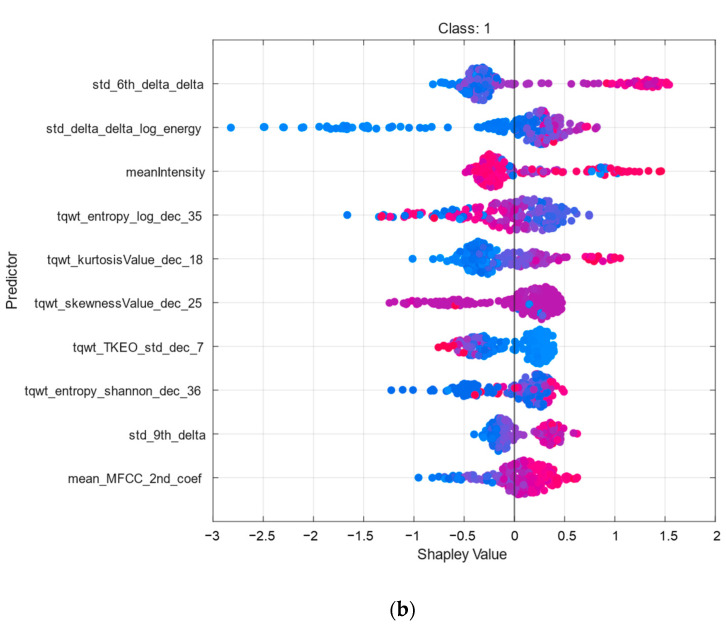
SHAP summary plots for GS-Ensemble model: feature contributions to PD classification for (**a**) Class 0 and (**b**) Class 1.

The SHAP summary plots in Figure 6 illustrate the feature contributions to the GS-Ensemble model, which achieved peak performance in PD classification with an accuracy of 90.27% and an F1-score of 89.94%. These plots reveal the most impactful features for each class (Class 0 and Class 1), highlighting the significance of wavelet-based measures. Features such as mean_MFCC_2nd_coef, which relate to the characteristics of acoustic signals, played a crucial role in the model’s decision-making process. The *x*-axis displays the SHAP values, showing the contribution of each feature in influencing a prediction toward a specific class. Meanwhile, the color gradient, ranging from yellow to blue, represents the feature values. These results highlight the GS-Ensemble model’s effectiveness in utilizing both time-frequency and acoustic features for precise classification.

## 4. Discussion

This study explored the application of machine learning algorithms for diagnosing PD using vocal characteristics. By leveraging a publicly available dataset, the study employed Chi-Square-based feature selection to identify the most relevant acoustic attributes and compared five different machine learning classifiers—SVM, k-NN, DT, NN, Ensemble and Stacking models. Additionally, three distinct hyperparameter optimization techniques—GS, RS, and BO—were evaluated to determine the most effective model configuration. The findings demonstrated that Stacking classifiers, particularly those optimized through GS, exhibited the highest performance, achieving 92.07% accuracy and an F1-score of 0.95. These results highlight the effectiveness of Stacking Learning and hyperparameter optimization in PD classification and confirm the potential of vocal characteristics as valuable biomarkers for early diagnosis.

Table 5 summarizes the accuracy, F1-score and AUC values from various studies on diagnosing PD using vocal characteristics. The GS-Ensemble approach proposed in this study yielded competitive results compared to existing methods, demonstrating a well-balanced performance across key metrics. While some studies have reported higher classification accuracies, the results of this study remain robust and consistent. For instance, while this study achieved 92.07% accuracy, prior works such as [40] (KNN, MLP, SVM and RF), [41] (MIRFE-XGBoost) and [42] (SVM cascaded DNN) have reported accuracies of 95.9%, 93.88%, and 96.42%, respectively. These variations may be attributed to differences in dataset size, preprocessing techniques, and modeling choices. However, it is important to emphasize that this study takes a holistic and interpretable approach, incorporating an extensive feature selection process and multiple optimization strategies to enhance generalization and model efficiency.

Despite the competitive performance of the GS-Ensemble and GS-Stacking models, further improvements can be explored. Future research could integrate evolutionary algorithms, such as genetic algorithms and particle swarm optimization, to refine hyperparameter selection. Additionally, hybrid architectures combining machine learning and deep learning approaches could further enhance feature representation and classification accuracy.

In summary, the proposed GS-Ensemble and GS-Stacking models achieve highly competitive results, particularly in terms of F1-score and AUC, while maintaining interpretability and computational efficiency. While certain deep learning models report marginally higher accuracy, this study provides a well-balanced and robust approach to PD classification using vocal characteristics. Future work will build on these efforts by fine-tuning Ensemble Learning strategies, implementing advanced optimization techniques, and increasing the diversity of datasets to increase classification accuracy and clinical relevance.

## 5. Conclusions

This paper aimed at exploring the behavior of a range of machine learning classifiers optimized with different hyperparameter tuning methods (BO, RS, and GS) for PD classification. To improve the efficiency and accuracy of the model, Chi-Square feature selection was used before training, which limited the classifiers to pivotal vocal features only. This preprocessing step enhanced the classification performance while keeping the computational complexity in check. It was observed that Stacking-based classifiers were superior to other models for all the optimization methods in terms of accuracy and F1-score. Among these, the GS-Stacking model was identified as the optimal strategy, which attained a test accuracy of 92.07% and F1-score of 0.95 compared to other classifiers in terms of predictive accuracy. These findings further reinforce the importance of feature selection and hyperparameter tuning to improve the performance of machine learning models for PD diagnosis. The effectiveness of Ensemble and Stacking Learning for complex classification tasks was demonstrated, especially in medical applications that involve vocal biomarkers, highlighting the GS-Stacking model’s success.

Although this study offered crucial findings in the application of machine learning for PD diagnosis, further investigations can build on these findings by examining new feature selection approaches, optimizing Ensemble Learning strategies, and assessing the effect of extra temporal vocal features on enhancing the diagnostic accuracy. Including more varied and more realistic clinical data in the dataset may also help to support the goodness of fit of the proposed model.

## Figures and Tables

**Table 1:** Hyperparameter ranges for machine learning models with BO, GS, and RS.

ML Classifiers	Parameter Name	Parameter Range
DT	Maximum number of splits (Mns)	1–529;
	Split criterion (Sc)	Gini’s diversity index, Maximum deviance reduction
SVM	Box constraint level (Bcl)	0.001–1000
	Kernel scale (Ks)	0.001–1000
	Kernel function (Kf)	Gaussian, Linear, Quadratic, Cubic
	Standardize data (Sd)	Yes, No
k-NN	Number of neighbors (Nn)	1–265
	Distance metric (Dm)	City block, Chebyshev, Correlation, Cosine, Euclidean, Hamming, Jaccard, Mahalanobis, Minkowski (cubic), Spearman
	Distance weight (Dw)	Equal, Inverse, Squared inverse
	Standardize data (Sd)	Yes, No
NN	Number of fully connected layers (Nfcl)	1–3
	Activation (Act)	ReLU, Tanh, Sigmoid, None
	Standardize data (Sd)	Yes, No
	Regularization strength (Lambda)	1.8868 × 10^−8^ –188.6792
	First layer size (Fls)	1–300
	Second layer size (Sls)	1–300
	Third layer size (Tls)	1–300
Ensemble	Ensemble method (Em)	Bag, GentleBoost, LogiBoost, AdaBoost, RUSBoost
	Number of learners (Nl)	10–500
	Learning rate (Lr)	0.001–1
	Maximum number of splits (Mns)	1–529

**Table 2:** Ranking of features based on Chi-Square scores.

Rank	Feature Group	Feature Name	Chi-Square Scores
1	Wavelet Transform	tqwt_TKEO_std_dec_12	47.5559
2	Wavelet Transform	tqwt_stdValue_dec_12	46.4329
3	Wavelet Transform	tqwt_TKEO_mean_dec_12	45.4982
4	Wavelet Transform	tqwt_entropy_shannon_dec_12	45.3114
5	Baseline	std_delta_delta_log_energy	45.2180
6	Wavelet Transform	tqwt_entropy_log_dec_12	42.7002
7	Wavelet Transform	tqwt_TKEO_std_dec_13	40.4690
8	Baseline	std_delta_log_energy	40.1906
9	Mel Frequency Cepstral Coefficients (MFCCs)	mean_MFCC_2nd_coef	39.7268
10	Wavelet Transform	tqwt_minValue_dec_12	36.0287
155	Wavelet Transform	tqwt_kurtosisValue_dec_20	20.1137
156	Baseline	std_7th_delta	20.0257
157–754	All	Other features	<20

**Table 3:** Optimal parameter values of ML classifiers obtained through three optimization methods.

ML Classifiers	Parameter Name	BO	GS	RS
DT	Mns	8	8	7
	Sc	Gini’s diversity index	Gini’s diversity index	Gini’s diversity index
SVM	Bcl	970.2948	215.4435	0.016299
	Ks	-	10	-
	Kf	Quadratic	Gaussian	Linear
	Sd	Yes	Yes	Yes
k-NN	Nn	12	3	2
	Dm	Correlation	Correlation	Correlation
	Dw	Squared inverse	Squared inverse	Inverse
	Sd	Yes	Yes	Yes
NN	Nfcl	3	2	1
	Act	None	Tanh	Sigmoid
	Sd	Yes	Yes	Yes
	Lambda	0.082873	4.065 × 10^−5^	9.6923 × 10^−7^
	Fls	207	159	15
	Sls	148	4	-
	Tls	85	-	-
Ensemble	Em	GentleBoost	GentleBoost	GentleBoost
	Nl	20	500	451
	Lr	0.61762	0.046416	0.0015096
	Mns	92	8	17

**Table 4:** Performance metric values of ML classifiers.

Data	Classifier	Accuracy	Error Rate	Precision	Recall	F1-Score
Training (Validation)	BO-DT	83.96	16.04	83.35	83.96	83.49
	BO-SVM	82.64	17.36	82.40	82.64	82.51
	BO-k-NN	86.60	13.40	86.40	86.60	86.48
	BO-NN	83.58	16.42	83.01	83.58	82.17
	BO-Ensemble	86.60	13.40	86.18	86.60	85.99
	BO-Stacking	87.52	12.48	89.97	92.84	91.38
	GS-DT	83.96	16.04	83.35	83.96	83.49
	GS-SVM	84.34	15.66	84.10	84.34	84.20
	GS-k-NN	86.23	13.77	86.49	86.23	86.34
	GS-NN	85.85	14.15	85.49	85.85	85.61
	GS-Ensemble	87.92	12.08	87.67	87.92	87.35
	GS-Stacking	89.04	10.96	91.21	93.63	92.41
	RS-DT	83.96	16.04	83.35	83.96	83.49
	RS-SVM	83.96	16.04	83.30	83.96	82.86
	RS-k-NN	86.23	13.77	86.41	86.23	86.31
	RS-NN	85.47	14.53	85.31	85.47	85.38
	RS-Ensemble	87.36	12.64	87.05	87.36	86.73
	RS-Stacking	86.77	13.23	88.86	93.10	90.93
Testing	BO-DT	79.20	20.80	79.58	79.20	79.38
	BO-SVM	84.07	15.93	83.42	84.07	83.53
	BO-k-NN	86.28	13.72	85.96	86.28	86.07
	BO-NN	82.30	17.70	81.49	82.30	80.53
	BO-Ensemble	86.28	13.72	85.80	86.28	85.65
	BO-Stacking	90.75	9.25	96.11	92.51	94.28
	GS-DT	79.20	20.80	79.58	79.20	79.38
	GS-SVM	86.73	13.27	86.58	86.73	86.65
	GS-k-NN	87.61	12.39	87.77	87.61	87.68
	GS-NN	86.73	13.27	86.31	86.73	86.38
	GS-Ensemble	90.27	9.73	90.10	90.27	89.94
	GS-Stacking	92.07	7.93	96.69	93.58	95.11
	RS-DT	80.09	19.91	80.71	80.09	80.36
	RS-SVM	84.96	15.04	84.55	84.96	83.78
	RS-k-NN	88.05	11.95	88.13	88.05	88.09
	RS-NN	84.96	15.04	84.33	84.96	84.20
	RS-Ensemble	89.38	10.62	89.14	89.38	89.02
	RS-Stacking	91.19	8.81	97.18	91.98	94.51

**Table 5:** Comparison of the literature.

References	Method	Accuracy	F1-Score	AUC
[40]	KNN, MLP, SVM and RF	95.9	-	-
[41]	MIRFE-XGBoost	93.88	93.74	0.978
[42]	SVM cascaded DNN	96.42	-	-
[43]	AdaBoost	96	95	-
[44]	Evolutionary Wavelet NNs	90	-	-
[22]	SVM	85	-	-
[45]	RF	99	96	-
[46]	Ensemble	95	97	-
[47]	Fine Tuned NN	86.47	-	-
[48]	Ensemble Voting	96.41	97.59	-
This study	GS-Ensemble	90.27	89.94	0.95
This study	GS-Stacking	92.07	95.11	0.95

## Data Availability

Publicly available data were used in this study. The data can be accessed from reference [25].

## Abbreviations

The following abbreviations are used in this manuscript:

PD	Parkinson’s Disease
MFCC	Mel Frequency Cepstral Coefficient
WT	Wavelet transform
RPDE	Recurrence Period Density Entropy
DFA	Detrended Fluctuation Analysis
PPE	Pitch Period Entropy
GQ	Glottis Quotient
GNE	Glottal to Noise Excitation
VFER	Vocal Fold Excitation Ratio
EMD	Empirical Mode Decomposition
BO	Bayesian Optimization
GS	Grid Search
RS	Random Search
TPR	True positive rate
FPR	False positive rate
AUC-ROC	Area under the receiver operating characteristic curve
SHAP	SHapley Additive exPlanations
SVM	Support Vector Machine
k-NN	k-nearest neighbor
DT	Decision Tree
NN	Neural Network
